# Origin, Succession, and Control of Biotoxin in Wine

**DOI:** 10.3389/fmicb.2021.703391

**Published:** 2021-07-22

**Authors:** Xiaoyu Xu, Tian Li, Yanyu Ji, Xia Jiang, Xuewei Shi, Bin Wang

**Affiliations:** School of Food Science and Technology, Shihezi University, Shihezi, China

**Keywords:** biogenic amines, biotoxin, ethyl carbamate, ochratoxin A, wine

## Abstract

Wine is a worldwide alcoholic beverage with antioxidant active substances and complex flavors. Moderate drinking of wine has been proven to be beneficial to health. However, wine has some negative components, such as residual pesticides, heavy metals, and biotoxins. Of these, biotoxins from microorganisms were characterized as the most important toxins in wine. Wine fermentation mainly involves alcoholic fermentation, malolactic fermentation, and aging, which endue wine with complex flavors and even produce some undesirable metabolites. These metabolites cause potential safety risks that are not thoroughly understood. This review aimed to investigate the origin, evolution, and control technology of undesirable metabolites (e.g., ochratoxin A, ethyl carbamate, and biogenic amines) in wine. It also highlighted current wine industry practices of minimizing the number of biotoxins in wine.

## Introduction

Wine is an alcoholic beverage made from fresh grapes or grape juice that undergoes complex biochemical changes in the presence of microorganisms. The wine originated in ancient Egypt or ancient Greece or the Greek island of Crete (Alebaki and Koutsouris, 2019). The wine industry has progressed globally since its development to date (Thorpe, 2009). Based on the geography, winemaking history, and winemaking tradition, some winemaking countries with a long history of production (mostly Europe and the Mediterranean region) are classified as “Old World,” while the rising stars in the international market are classified as “New World” (emerging wine-producing countries outside of Europe, such as the United States, China, etc.) (Banks and Overton, 2010; Li et al., 2018). The three leading wine-producing countries worldwide are France, Italy, and Spain, which produce almost half of the world’s wine (Schamel, 2006). According to the latest data from the International Organization of Vine and Wine (OIV), global wine production is estimated at 26 billion liters, and the wine trade continues to trend toward internationalization (OIV, 2020).

Nowadays, wine is attracting an increasing amount of attention due to its taste, aroma, and health benefits (Ditano-Vázquez et al., 2019; Rivera et al., 2019). While exploiting the various benefits of wine, its quality is often easily overlooked. Similar to other fermented foods, the fermentation process of wine creates a complex system of grape flavors and may also present some quality risks, such as heavy metals, pesticide residues, and biotoxins (Weng and Neethirajan, 2017). Among these, ethyl carbamate (EC) from yeast and lactic acid bacteria (LAB) (Uthurry et al., 2006; Du et al., 2018), biogenic amines (BAs) from LAB (García-Ruiz et al., 2011), and ochratoxin A (OTA) from mold (Iacumin et al., 2009) have gradually received attention in recent years. EC was shown to be a carcinogen as early as 1943 (Nettleship et al., 1943), and alcohol contributes to the carcinogenic effects of EC (Beland et al., 2005). BAs are also precursors to carcinogens (Guo et al., 2015), and hence their excessive intake can pose a threat to human health.

The production of high-quality wine has stringent requirements for grape raw materials (Morata et al., 2019), ferments, and grape processes, but their potential safety risks cannot be ignored. The risk factors of OTA, EC, and BAs have been identified in wine one after another; however, the sources of these risk factors and their evolution patterns are still unclear. This review focused on the dynamic changes in risk factors in wine fermentation, traced the risk factors, and proposed corresponding prevention and control to provide a theoretical basis for wine risk control.

## Safety Risks and Countermeasures in Wine

**FIGURE 1:**
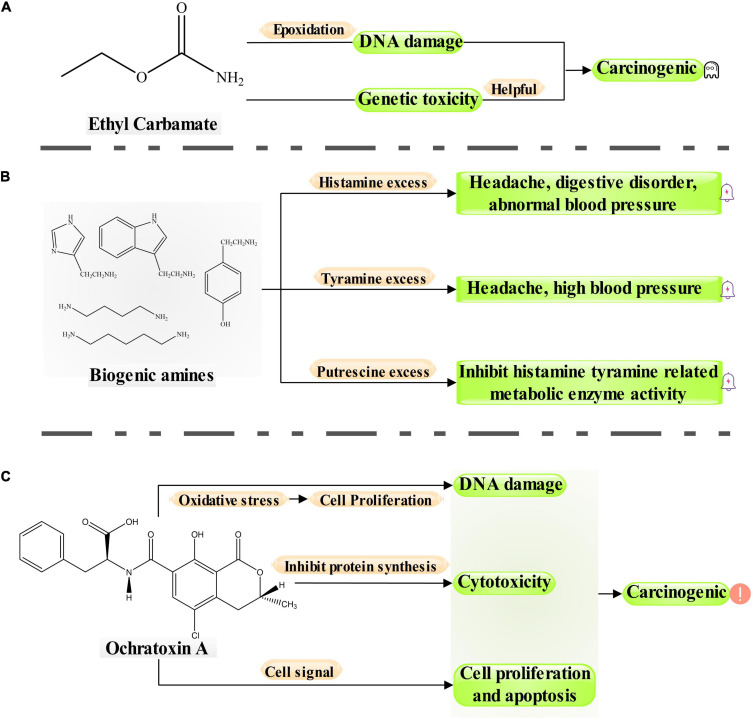
Hazard process of biologically harmful products (ethyl carbamate, biogenic amines and ochratoxin A) in wine.

**FIGURE 2:**
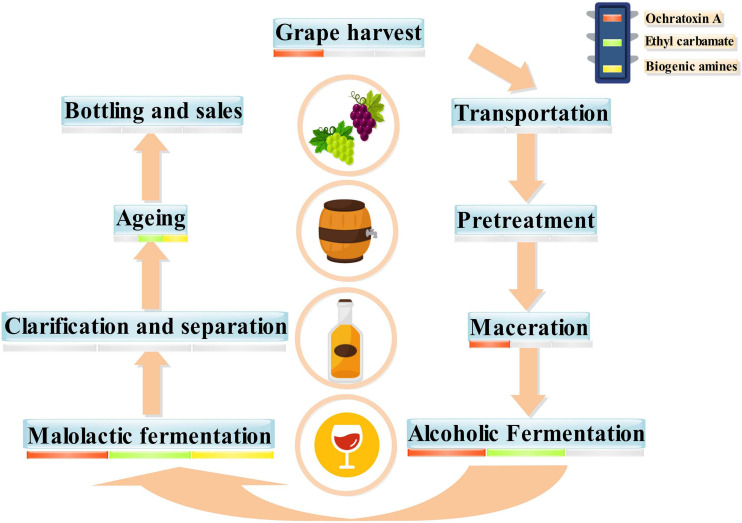
Winemaking process and possible safety hazards.

Moderate drinking of wine has been proven to be beneficial to health because wine comprises antioxidant active substances, minerals, and vitamins (Guilford and Pezzuto, 2011). However, wine can also have some negative components, such as residual pesticides (Guo T. et al., 2016), heavy metals (Bora et al., 2015), and some biotoxins. Of these, biotoxins from microorganisms were the most important toxins in wine (Vitali Čepo et al., 2018). These biotoxins can affect the drinking quality and food safety of wine and lead to a range of diseases if consumed in excess over a long period (Figure 1; Welke, 2019). The biotoxins of microbial origin in wine mainly comprise OTA, EC, and BAs. The process from grapes to wines is long and complex, including transportation, pretreatment, maceration, and alcoholic fermentation (Ruiz et al., 2019). During wine fermentation, OTA, EC, and BAs undergo continuous evolution (Christaki and Tzia, 2002; Fernández-Segovia et al., 2014). Grape harvesting, maceration, alcoholic fermentation, and malolactic fermentation (MLF) involve the production of OTA. EC is always produced in alcoholic fermentation, MLF, and aging. Furthermore, various BAs are formed during MLF and aging (Figure 2).

### Ochratoxin A

Ochratoxin is a mycotoxin composed of seven structurally similar compounds, including OTA, OTB, and OTC (Supplementary Figure 1). Among these, OTA is an IIB carcinogen, which has teratogenicity, nephrotoxicity, hepatotoxicity, neurotoxicity, and immunotoxicity to several kinds of animals (Silva et al., 2019). The OTA biosynthesis and the two possible key pathways involved are shown in Supplementary Figure 2 (Karlovsky, 1999; Gallo et al., 2017). OTA has attracted much attention because of its strong biological toxicity and potential pathogenicity in various cereal crops and fermented foods (Agriopoulou et al., 2020). As far as 1996, OTA was first identified in wine and then classified as the key mycotoxin in wine (Zimmerli and Dick, 1996). The European Commission set the maximum limit for OTA content in wine at 2 μg/kg (European Food Safety Authority (EFSA), 2006).

OTA is produced by various mycetes, including *Aspergillus ochraceus*, *Penicillium verrucosum*, *Aspergillus niger*, and *Aspergillus carbonarius*. However, the main fungal sources of OTA in grapes are *A. carbonarius* and *A. niger* (Oliveri et al., 2017). *A. carbonarius* has been considered as the most important ochratoxin-producing species in grapes because it is widespread on grapes and produces a high concentration of OTA (Varga and Kozakiewicz, 2006). Like other mycetes, *Aspergillus* spp. can produce spores, which are blown to the surrounding grape racks by wind and flying animals (Jiang et al., 2013). During the ripening of grapes, humid weather and high relative humidity can easily cause the rotting of grapes by providing favorable conditions for the growth of *Aspergillus* spp. (Cañas et al., 2008). Obviously, the environmental conditions of a vineyard play a key role in the contamination of ochratoxin-producing species in wine grapes, further leading to the accumulation of OTA in wine (Gil-Serna et al., 2018; Abarca et al., 2019). Before wine fermentation, the grape skin and pulp are crushed and macerated together, which is conducive to the release of OTA into the grape juice (Visconti et al., 2008). The OTA content changes greatly in the whole winemaking process (Anli and Bayram, 2009; Freire et al., 2020). Due to the different winemaking processes, red wines generally have higher OTA levels compared with white wines (Lasram et al., 2008; Dachery et al., 2017).

Some methods, such as avoiding mycete infection, degrading OTA, and adsorbing OTA, have been developed to decrease the contamination of OTA (Chen et al., 2018). Applying biological control methods to avoid mold infection during the storage of grapes after harvest and removing moldy grape clusters before fermentation can greatly reduce the possibility of toxin-producing fungal growth and production of OTA (Hocking et al., 2007; Gil-Serna et al., 2018). Inorganic adsorbents (such as zeolite and activated carbon) (Piotrowska et al., 2013; Abrunhosa et al., 2014) and microbial adsorbents (such as *Saccharomyces* spp., *Rhodotorula* spp., *Lactobacillus* spp., and *Cryptococcus* spp.) (Abrunhosa et al., 2010; Russo et al., 2016) reduce the OTA content through adsorbing or converting OTA into less toxic phenylalanine (Phe) and ochratoxin alpha (OTα). However, the application of these adsorbents in OTA control in wine is limited because they can adsorb phenolic compounds and pigments of wine to varying degrees, resulting in wine discoloration, besides adsorbing the risky OTA (Caridi, 2013; Petruzzi et al., 2015). Moreover, microbial-derived enzymes with carboxypeptidase A activity also affect the degradation of OTA (Amézqueta et al., 2009).

### Ethyl Carbamate

As early as 1943, EC was classified as a Class 2A carcinogen by the International Agency for Research on Cancer of the World Health Organization (2007) (Conacher and Page, 1986; Zimmerli and Schlatter, 1991). EC is a carcinogenic compound involved, among others, in lung cancer, lymphoma, liver cancer, and skin cancer (Gowd et al., 2018). However, it is believed that EC widely occurs in traditional fermented foods (Li and Bardají, 2017). EC has been recognized as one of the biggest challenges facing the alcoholic beverage industry since EC was detected in alcoholic beverages in 1976 (Ough, 1976; Zhao et al., 2013a). Supplementary Figure 3 shows the possible pathways of the formation of EC and the mechanism of carcinogenesis (Zhao et al., 2013a). Different countries and organizations around the world have different standards for the concentration of EC in alcoholic beverages (Supplementary Table 1). Also, no unified maximum EC limit exists in the EU. However, the concentration of EC in 30.6% of wines exceeds 20 μg/L (standard of the Food and Agriculture Organization of the United Nations), which is a threat to the health of consumers and the sustainable development of the wine industry (Gowd et al., 2018).

EC is generally produced by the spontaneous reaction of ethanol and compounds containing carbamoyl groups (such as urea, citrulline, carbamoyl phosphate, and so on) (Jiao et al., 2014). Among these reactions, the urea formation pathway is believed to be the main formation pathway of EC (Zimmerli and Schlatter, 1991; Cerreti et al., 2016). Wine environments (such as temperature and acidity) and microorganisms can affect the production of EC during fermentation (Zhao et al., 2013a). During grape plantation, the application of nitrogen fertilizer increased the urea content, providing EC precursors in grapes (Garde-Cerdán et al., 2015). Furthermore, yeast and LAB produced a large amount of citrulline through the urea cycle pathway and the arginine deiminase metabolism pathway, respectively (Azevedo et al., 2002; Vrancken et al., 2009). During wine fermentation, some EC precursors have been released, increasing the urea content in wine (Mira de Orduña et al., 2000). The EC content in wine varied with grape varieties (Ubeda et al., 2020), grape maturity (Lago et al., 2017), pH value (Araque et al., 2013), EC precursor concentration (Zhao et al., 2013b), the volume fraction of ethanol (Araque et al., 2013), and ecological conditions (such as temperature, precipitation, and extreme climate) (Diamantidou et al., 2018).

Controlling the EC content in wine mainly focuses on yeast strains (Araque et al., 2013; Guo T. et al., 2016), grapes (Bell and Henschke, 2005), excipients (Bell and Henschke, 2005), and fermentation conditions (Stevens and Ough, 1993; Xue et al., 2015). The enhancement of genes encoding for enzymes involved in urea degradation and transport or the knockout of genes encoding for arginase allowed the selection of yeast strains with low urea production capacity and arginase activity (Araque et al., 2013; Guo X. W. et al., 2016). Properly adjusted vineyard management practices, such as fertilization, pruning, irrigation, and ground cover, can also control the EC content in wine to some extent (Soufleros et al., 2003). Without affecting the flavor of the wine, an appropriate reduction in temperature also helps reduce the EC content in the wine, which is a key adjustment point for EC control from a process perspective (Hasnip et al., 2004). Some studies showed that acid urease catalyzed the decomposition of urea to ammonia and carbon dioxide, decreasing the content of an important precursor of EC in wine (Cerreti et al., 2016; Liu et al., 2018; Yang et al., 2021). Since 1999, Europe has approved the use of acid urease extracted from fermented LAB in wine (Cerreti et al., 2016). However, urease is a metalloenzyme with nickel as a prosthetic group, which can lead to nickel residues in wine (Follmer et al., 2004). Furthermore, urea adsorbents, EC degrading enzymes, EC adsorbents, and so forth have been used as effective and potential agents to controlling the EC content in wine under the premise of ensuring the flavor characteristics of the original wine (Wu et al., 2014; Zhou et al., 2017).

### Biogenic Amines

BAs are a class of low-molecular-weight nitrogen-containing organic compounds (Manetta et al., 2016). A physiological concentration of BAs is involved in important biological reactions in the human body (Cinquina et al., 2004). However, the excessive intake of exogenous BAs can lead to allergic reactions, such as headache, nausea, blood pressure changes, and respiratory disorders, and is even life-threatening (Spano et al., 2010). BAs include mainly tryptamine, cadaverine, tyramine, histamine, putrescine, spermidine, and spermine (Supplementary Table 2), which are usually produced after decarboxylation of the corresponding amino acids by different decarboxylases (Supplementary Figure 4; Wolken et al., 2006). As the most toxic BAs, histamine is regarded as a key indicator of the hygienic value during wine fermentation (Cunha et al., 2017). It can be broken down by two different oxidase enzymes (monoamine oxidase and diamine oxidase) (Seiler, 2004). However, ethanol is an inhibitor of diamine oxidase (histamine-degrading) at the gut level (García-Ruiz et al., 2011). Putrescine has been found to be one of the most abundant BAs in wine (Henríquez-Aedo et al., 2012; Cunha et al., 2017). Therefore, the BA content is more stringent in alcoholic foods compared with other fermented foods (Rollan et al., 1995; García-Ruiz et al., 2011). The European countries have set limits for histamine below 10 mg/L in wine, with Germany being the strictest (not higher than 2 mg/L) (Smit et al., 2008; Costantini et al., 2019).

BAs are produced by microorganisms with amino acid decarboxylase activity in the presence of sufficient free amino acids at any stage of winemaking (Santos, 1996). The presence of precursors (amino acids) and microorganisms with decarboxylase activity are the main factors affecting the BA content (Landete et al., 2011; Russo et al., 2016). During winemaking, yeast strains weakly contribute to BA accumulation (Smit et al., 2013), but LAB responsible for MLF has been identified as the main producer of BAs in wine (Spano et al., 2010). For example, *Oenococcus oeni*, *Lactobacillus hilgardii*, and *Pediococcus parvulus* increased the BA content in wine through histamine accumulation (Özogul and Hamed, 2018). As one of the main catabolites from arginine degradation, putrescine has been identified as one of the most abundant BAs in wine, because arginine is the main amino acids in grapes (Henríquez-Aedo et al., 2012; Ortega-Heras et al., 2014; Cunha et al., 2017). *O. oeni* also contributes to putrescine accumulation after sequential degradation of arginine and ornithine (Pessione and Cirrincione, 2016). On the other hand, increasing pH can increase the number and variety of microorganisms, further enhancing the risk (Guo et al., 2015). The BA content in white wines is less than that in red wines because of a lower pH and the different winemaking processes (García-Marino et al., 2010).

The production of BAs is a strategy to obtain metabolic advantages to face certain stress conditions. Therefore, conditions encouraging the expression of decarboxylase genes should be avoided and controlled (Mohedano et al., 2015). Moreover, commercial starters with negative decarboxylase activity are also recommended (Gardini et al., 2016). Several authors have proposed that yeast can convert amino acids into fused alcohols through the well-known Ehrlich pathway during alcoholic fermentation, plausibly leading to a decrease in the BA content (Mas et al., 2014). In a sense, the presence of putrescine in wine seems inevitable, because the ornithine decarboxylation occurs simultaneously with MLF at a high speed (Martínez-Pinilla et al., 2013; Battistelli et al., 2020). Due to the presence of the indigenous strains capable of degrading arginine to ornithine, the use of the malolactic starters that are unable to degrade ornithine or arginine cannot completely avoid accumulation of putrescine (Pramateftaki et al., 2012). It is plausible that removing or inhibiting the activity of LAB immediately after MLF to avoid arginine degradation may be an effective method to reduce potential risk from putrescine in wine (Wunderlichová et al., 2014). Therefore, controlling microbiota is a good strategy to reduce BA production. García-Ruiz et al. (2011) found that nine strains belonging to the *Lactobacillus* and *Pediococcus* groups showed the greatest BA degradation capacity, the best being for *L. casei* IFI-CA52. Capozzi et al. (2012) have investigated that *Lactobacillus plantarum* NDT 09 and NDT 16 could enhance the overall aroma of wine and degrade putrescine and tyramine. Some yeasts were also capable of degrading BAs. Bäumlisberger et al. (2015) observed that some strains of *Debaryomyces hansenii* and *Yarrowia lipolytica* could degrade BAs. The degradation of BAs by the most efficient strain, *D. hanseniii* H525, could be attributed to a peroxisomal amine oxidase activity. Callejón et al. (2016) reported the employment of laccase to degrade BAs, which provides a new perspective on the use of microorganisms or purified microbial enzymes. Further research should be conducted to find new strains capable of degrading BAs. Also, histamine, putrescine, cadaverine, spermine, and spermidine in wine can be adsorbed and removed by phosphonic acid and sulfonic acid bifunctional mesoporous silica materials, which may also be an effective way to reduce the BA content in wine in the future (Rodríguez-Bencomo et al., 2020).

## Conclusion and Future Perspectives

The quality and safety risks of wine involve many links. To ensure food safety and improve the quality of wine, we need to control the contamination of raw materials, strictly select winemaking microorganisms, and control the fermentation and post-management processes. The study of the origin, evolution, and control techniques of undesirable metabolites in wine (OTA, EC, and BAs) can reduce not only the quality hazards of wine but also the economic losses due to microbial spoilage. In the future, we should pay attention to various potentially harmful substances that have pathogenic effects on human beings during the grape growth and winemaking process, and implement effective prevention and control through testing. In summary, only by clarifying the factors that affect the quality of winemaking can we ensure a clear direction for quality management and ultimately a quality wine production.

## Author Contributions

XX wrote the main manuscript. TL and YJ conceived the framework of the manuscript. XJ contributed to the pictures in the manuscript. XS and BW coordinated contributions and provided the final draft of the manuscript. All authors commented on the manuscript at all stages.

## Conflict of Interest

The authors declare that the research was conducted in the absence of any commercial or financial relationships that could be construed as a potential conflict of interest.
