# Supplementary material for: Origin, Succession, and Control of Biotoxin in Wine
Source: Front Microbiol. 2021 Jul 22;12:703391. doi: 10.3389/fmicb.2021.703391 (PMC8339702; doi:10.3389/fmicb.2021.703391)
Supplement: Supplementary file 1 [file Data_Sheet_1.docx]

Supplementary materials for:

**Origin, Succession and Controlment of Biotoxin in Wine** Xiaoyu Xu, Tian Li, Yanyu Ji, Xia Jiang,Xuewei Shi*, Bin Wang*

Food College, Shihezi University, Shihezi 832000, Xinjiang Uygur Autonomous Region, P. R. China.

* Corresponding authors

E-mail addresses: B. W.: [binwang0228@shzu.edu.cn](mailto:binwang0228@shzu.edu.cn); X. S.: [shixuewei@shzu.edu.cn](mailto:shixuewei@shzu.edu.cn)

Tel.: 86-0993-2058093

CATALOGUE

[**Supplementary tables 1**](#_Toc75229662)

[Table S1 Maximum residue limits for EC in alcoholic beverages 1](#_Toc75229663)

[Table S2 Common BAs and their molecular weight, molecular formula and chemical structure in wine 2](#_Toc75229664)

[**Supplementary figures 3**](#_Toc75229665)

[Figure S1 OTA main conjugation and degradation products. 3](#_Toc75229666)

[Figure S2 Key steps of OTA biosynthesis and biodegradation pathways. 4](#_Toc75229667)

[Figure S3 Formation of EC and its carcinogenic pathways. 5](#_Toc75229668)

[Figure S4 Biogenesis of BAs and histamine degradation pathways. 6](#_Toc75229669)

[**References 7**](#_Toc75229670)

# Supplementary tables

## Table S1 Maximum residue limits for EC in alcoholic beverages

| Country and organization | EC concentration of wine(μg/L) | EC concentration of fortified Wine（μg/L） | EC concentration of fruit Brandy（μg/L） | EC concentration of sake（μg/L） | EC concentration of distilled Liquors（μg/L） | References |
| --- | --- | --- | --- | --- | --- | --- |
| America | 15 | 60 | - | - | - | (European Food Safety, 2007) |
| France | - | - | 1000 | - | 150 | (European Food Safety, 2007) |
| Germany | - | - | 800 | - | - | (European Food Safety, 2007) |
| Czech Republic | 30 | 100 | 400 | 200 | 150 | (European Food Safety, 2007) |
| Canada | 30 | 100 | 400 | 200 | 150 | (European Food Safety, 2007) |
| Japan | 30 | 100 | 400 | 100 | 150 | (Weber and Sharypov, 2009) |
| Korea | 30 | - | - | - | - | (Lee, 2013) |
| Brazil | - | - | - | - | 150 | (Lachenmeier et al., 2010) |
| Switzerland | - | - | 1000 | - | - | (Weber and Sharypov, 2009) |

^1^Symbol “-” represents that there is no stipulation so far.

## Table S2 Common BAs and their molecular weight, molecular formula and chemical structure in wine

| Number | Compound | Molecular weight | Molecular formula | Chemical structure |
| --- | --- | --- | --- | --- |
| 1 | Histamine | 111.1 | C_5_H_9_N_3_ |  |
| 2 | Tryptamine | 160.2 | C_10_H_12_N_2_ |  |
| 3 | Phenethylamine | 121.2 | C_8_H_11_N |  |
| 4 | Tyramine | 137.2 | C_8_H_11_NO |  |
| 5 | Spermidine | 145.2 | C_7_H_19_N_3_ |  |
| 6 | Spermin | 202.3 | C_10_H_26_N_4_ |  |
| 7 | Cadaverine | 202.2 | C_5_H_14_N_2_ |  |
| 8 | Putrescine | 88.2 | C_4_H_12_N_2_ |  |

# Supplementary figures


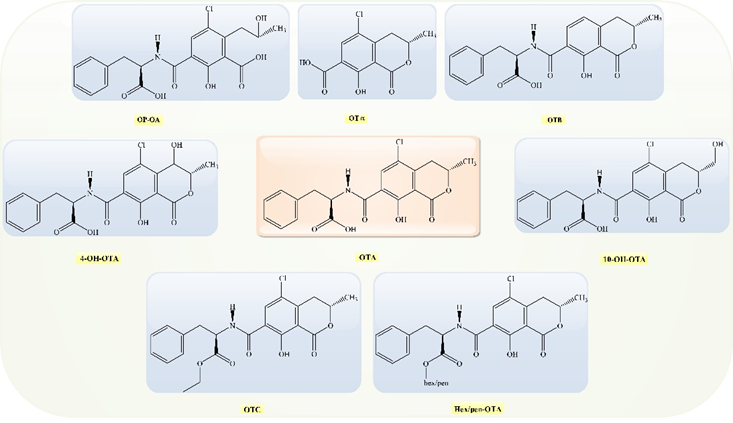


## FigureS1OTA main conjugation and degradation products.


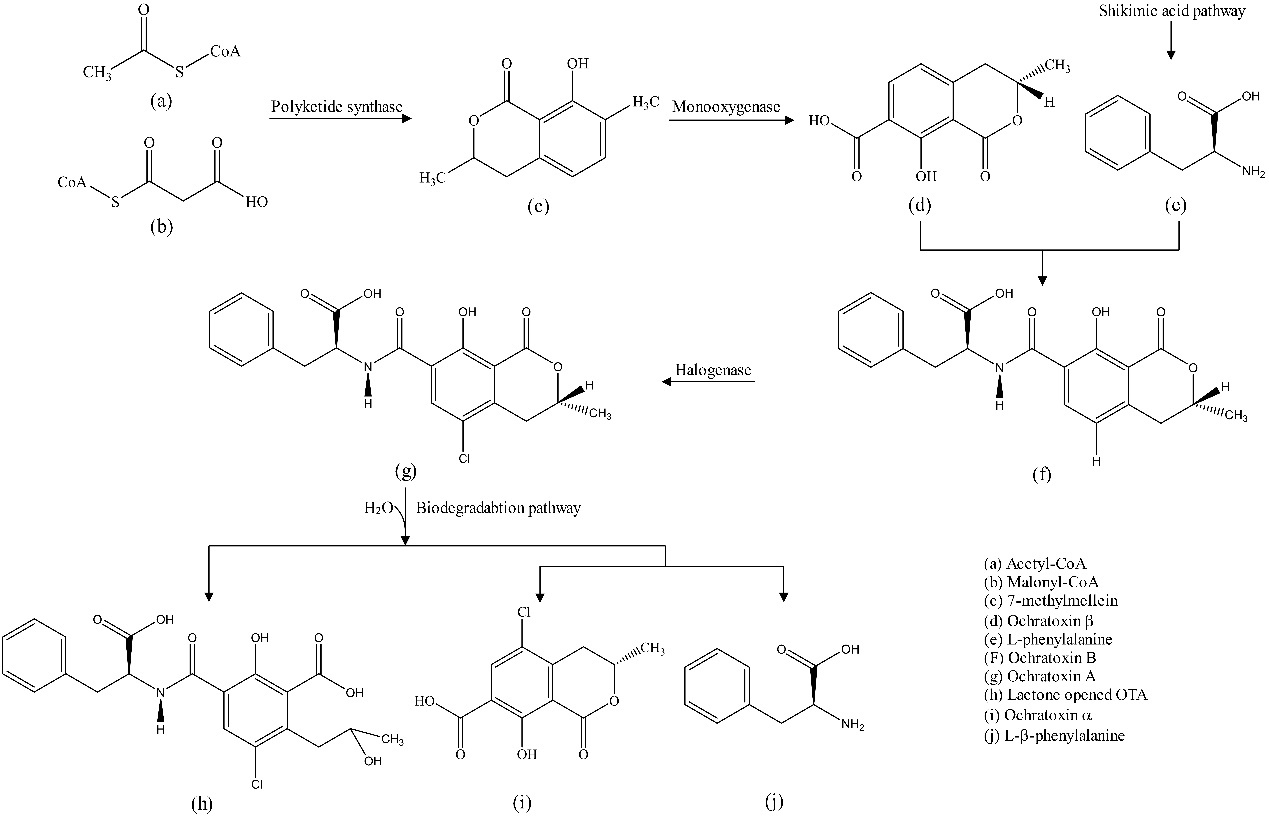


## FigureS2Key steps of OTA biosynthesis and biodegradation pathways.


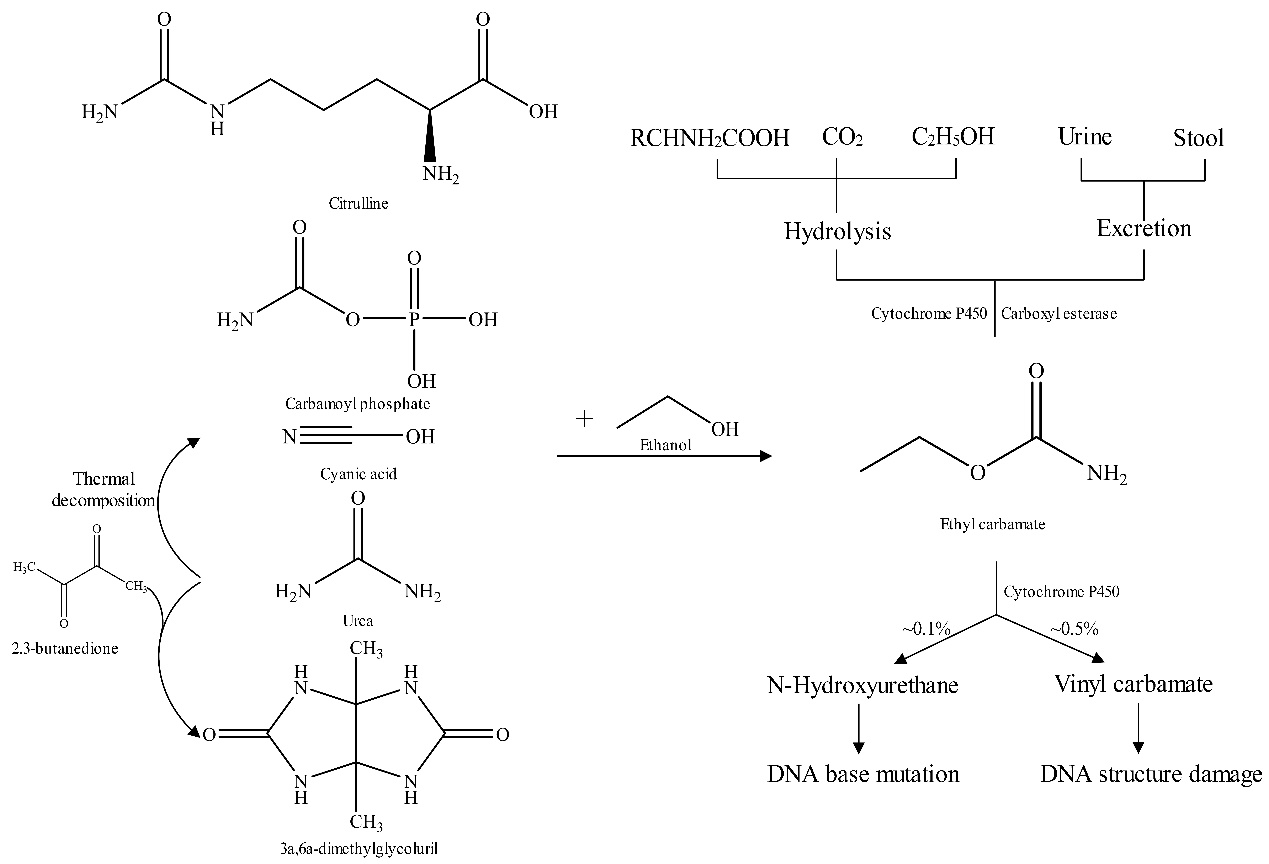


## **FigureS3 Formation of EC and its carcinogenic pathways.**The main precursors and pathways of EC production are described. Subsequently, most of the EC can be hydrolyzed or excreted by the human body. However, about 0.1% of EC can be converted into N-hydroxyurethane, and 0.5% can be converted into Vinyl carbamate and other substances, both of which may cause cancer.


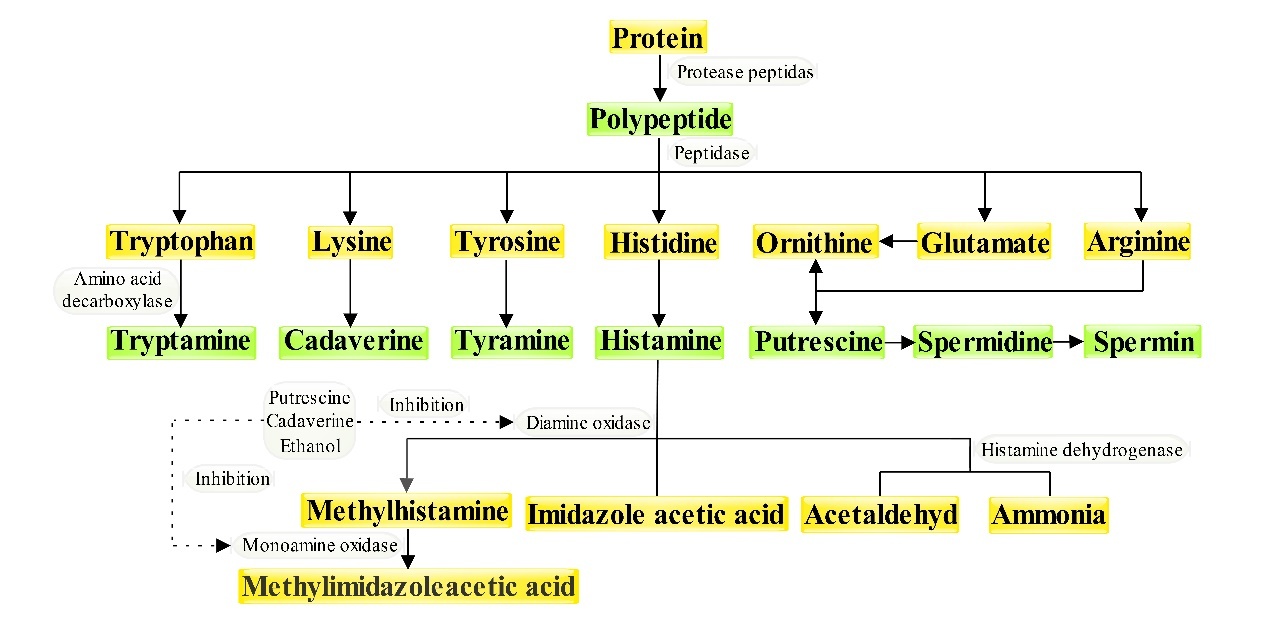


## **FigureS4 Biogenesis of BAsand histamine degradation pathways.**Cadaverine and putrescine can inhibit the activity of histaminerelated metabolic enzymes (e.g., monoamine oxidase and diamine oxidase) and ethanol is also an inhibitor of amine oxidase, both of which can increase histamine levels.

# References

European Food Safety, A. (2007). Ethyl carbamate and hydrocyanic acid in food and beverages - Scientific Opinion of the Panel on Contaminants. *EFSA Journal*. 5, 551.doi:10.2903/j.efsa.2007.551

Lachenmeier, D.W., Lima, M.C.P., Nóbrega, I.C.C., Pereira, J.A.P., Kerr-Corrêa, F., Kanteres, F., et al. (2010). Cancer risk assessment of ethyl carbamate in alcoholic beverages from Brazil with special consideration to the spirits cachaça and tiquira. *BMC Cancer.* 10,266. doi: 10.1186/1471-2407-10-266

Lee, K.G. (2013). Analysis and risk assessment of ethyl carbamate in various fermented foods. *Eur. Food Res. Technol.* 236, 891-898. doi: 10.1007/s00217-013-1953-6

Weber, J.V., and Sharypov, V.I. (2009). Ethyl carbamate in foods and beverages: a review. *Environ. Chem. Lett*. 7, 233-247. doi:10.1007/s10311-008-0168-8
